# Quantifying long-range correlations and 1/*f* patterns in a minimal experiment of social interaction

**DOI:** 10.3389/fpsyg.2014.01281

**Published:** 2014-11-12

**Authors:** Manuel G. Bedia, Miguel Aguilera, Tomás Gómez, David G. Larrode, Francisco Seron

**Affiliations:** Department of Computer Science and Engineering Systems, University of ZaragozaZaragoza, Spain

**Keywords:** perceptual crossing, social engagement, long-term correlations, multiscale interaction, 1/*f* noise, multifractality

## Abstract

In recent years, researchers in social cognition have found the “perceptual crossing paradigm” to be both a theoretical and practical advance toward meeting particular challenges. This paradigm has been used to analyze the type of interactive processes that emerge in minimal interactions and it has allowed progress toward understanding of the principles of social cognition processes. In this paper, we analyze whether some critical aspects of these interactions could not have been observed by previous studies. We consider alternative indicators that could complete, or even lead us to rethink, the current interpretation of the results obtained from both experimental and simulated modeling in the fields of social interactions and minimal perceptual crossing. In particular, we discuss the possibility that previous experiments have been analytically constrained to a short-term dynamic type of player response. Additionally, we propose the possibility of considering these experiments from a more suitable framework based on the use and analysis of long-range correlations and fractal dynamics. We will also reveal evidence supporting the idea that social interactions are deployed along many scales of activity. Specifically, we propose that the fractal structure of the interactions could be a more adequate framework to understand the type of social interaction patterns generated in a social engagement.

## 1. Introduction

There are emergent social processes in collective online situations—when two persons are engaged in real-time interactions—that can not be captured by a traditional offline perspective, whereby the problem is considered from the perspective of an isolated individual who acts as an observer exploiting their internal cognitive mechanisms to understand people. Although the study of how people process social information can be considered an old problem, in recent years, social cognitive processes have generated significant interest. On the one hand, theoretical interest, for example, a promising theoretical proposal has been developed about the possible “constitutive” role of social interaction for social cognition (De Jaegher, 2009; De Jaegher et al., 2010) that suggests that interactivity capabilities of the “second-person perspective” (Gomila, 2013) are the basis that support the “first and the third person approaches” and their related structure of mental states (Reddy, 2008; Wilms et al., 2010). On the other side, experimental interests; for example, the recent development of a minimal and simple framework for studying social online interactions, and for understanding the mechanisms that give support to minimal social capabilities (Auvray et al., 2009) that is known as the “perceptual crossing framework.” This experimental frame is a way to study online dyadic interactions and to analyze the perception of someone else's agency in different situations implemented in a minimal virtual world. Through the self-organized collective patterns that emerge from the interactions (like emergent coordination, turn-taking, etc.), hypotheses about the human capacity for social cognition can be extracted.

Perceptual crossing paradigm constitutes a simple framework for studying social online interactions in its simpler form. It consist of a minimal scenario in which two participants, sitting in different rooms, interact each other by moving a sensor along a shared virtual line using a computer mouse. The subjects are only allowed to move laterally in a one-dimensional world and perceive the collisions with other human subjects or artificial agents. In the last few years, the perceptual crossing paradigm has become a promising experimental tool for the analysis of dynamic interactions of human social processes. A more detailed analysis leads to two types of experiments: (i) behavioral experimental research and (ii) simulated agent modeling. Relating to the former, numerous experiments where real subjects try to identify each other in a virtual world have been carried out, and researchers have analyzed the type of behaviors that seem to offer support for social coordination patterns [for example, in one-dimensional experiments (Auvray et al., 2009) and also in their extensions to two dimensions (Lenay et al., 2011)]. In some cases, real experiments and phenomena previously tested in simulations were combined, for example in Iizuka et al. (2009, 2012), where authors explored how participants modulated the interaction dynamics to figure out if an interaction was live or not. Regarding the latter, i.e., focusing now on the computational modeling context (for example in Iizuka and Paolo, 2007; Iizuka et al., 2012), virtual agents have been evolved to locate others in an experimental set-up analogous to that used in Auvray's version (Auvray et al., 2009), providing a mathematical analysis that explained how virtual agents managed their own variables, such as size or velocity, to coordinate with others in an extremely robust way. Simulation models to build “social software agents” have demonstrated that this kind of behavior can emerge from very simple structures without explicit social reasoning capabilities (Froese et al., 2014a). In general, the studies on simulation models complimented the experimental work with humans, sometimes providing proofs of concept and a methodological alternative to explore social interactions.

A common feature exists in the way in which we deal with the experimental results obtained in both cases cited (considering behavioral modeling experiments and simulated agents): the participants' behavior is analyzed only *in a short-time scale* (this point is explained in detail in the next section). As how later, in this paper we propose to get a quantitative indicator that works as a complementary measure of the analysis addressed in previous perceptual crossing experiments, an indicator that consists of characterizing the cross-scale nature of the interaction through *fractal and multifractal analysis* (Van Orden et al., 2003) of the collective dynamics. We argue that this indicator can help to shed some light on the understanding of social constitutive processes and related questions and will be useful in order to characterize the *genuine constitution of social interactions*.

This is a brief outline of the paper: in Section 2, our working proposal is detailed and we propose that a multiscale analysis is needed to identify the type of pattern that emerges in a social interaction. Notions of 1/*f* patterns and fractal measures are explained in order to support the idea that “1/*f* noise analysis” (Van Orden et al., 2003, 2005) can be a genuine indicator able to discriminate between “human-human” or “human-software agent” interactions in a perceptual crossing experiment. In Section 3, we explain the type of experiments that we have carried out and the fractal and multifractal analysis on the results obtained. We also deliberate whether or not our results imply new insights into the characterization of social interactions. In Section 4, we discuss whether or not the results are statistically significant. Finally, Section 5 includes a review of the most notable points related to our analysis and future lines of research to be explored. An Appendix of Supplementary Material are included at the end of the paper. The first S.1 relates to the software platform and the protocols used for the experiments. The second focuses on the statistical foundations that give support to the results obtained.

## 2. Theoretical framework

Studies of the perceptual crossing experiment have provided insightful evidence about the importance of inter-individual coordination for the emergence of social cognition and agency detection. However, we think that still more advances are needed in order to characterize and better understand how coordinated interactions may give rise to collective social processes. Recently, some authors have emphasized the importance of understanding how distinct time scales and organizational levels are intertwined for the emergence of social cognition (Dumas et al., 2014). At neural level, there is experimental evidence of the importance of non-linear cross-scale interactions for brain organization ((Le Van Quyen, 2011), and in social neuroscience, inter-brain synchronization in multiple frequency bands has been found during imitation of hand movements (Dumas et al., 2010) or synchronization patterns are found during guitar improvisation showing a complex interplay of different frequencies (Müller et al., 2013). It is still missing to our knowledge a detailed analysis of this kind of phenomena at a behavioral level. These examples show the potential of a multi-scale account of social cognition and lead us to think that sometimes the analyses developed so far to understand perceptual crossing dynamics may fall short in their ability to characterize the emergent multi-scale nature of social interaction.

As previously stated, we contend that some of the conclusions about the original perceptual crossing experiments only focus on the reaction to short-term interactions. For example, in Auvray et al. (2009) the analyzed is limited to analyzing the probability of clicking in a 2 s window after the subject encounters another subject or object and by standard statistical variables of some system variables (frequency of crossings, correlation between velocity and acceleration, etc.). Thus, it is implicitly assumed that the emergence of social engagement can be reduced to a scale of short-term activity and that there is no influence of other scales or any inter-scale correlations that are relevant for the subject behavior (for example, assuming that there is no interference between the previous collisions of the subject with different kinds of agents and the decision of clicking or not clicking). A similar assumption is also found in the agent modeling field, for example in Di Paolo et al. (2008), where the simulated model is focused on finding what kind of short term dynamics (modeled in terms of delays between the perceptual stimulation of the agent and its motor response) is able to create the stable pattern of social interaction that allows a dynamic of co-regulation to emerge. Again, inter-scale correlations in the social interaction process are left out of the analysis and modeling.

In this context, we propose that it may be useful to analyze the dynamics in the perceptual crossing experiment from a conceptual framework that is not constrained by the assumption of one dominating scale of behavior. Despite its apparent simplicity, we propose that the perceptual crossing paradigm could comprise several embedded levels of dynamic interaction, resulting in correlations of the signals over different time scales. Therefore, in the next section we propose a framework of analysis that is capable of capturing the multiple relations between different scales of behavior. Specifically, in this next section we propose the analysis of fractal and multifractal patters for obtaining evidence of the multi-scale nature of social interaction in the perceptual crossing experiment.

### 2.1. 1/*f* noise and multifractality for characterizing social interaction

During the last two decades, the 1/*f* noise approach to cognitive science has achieved considerable progress in toward conceptualizing cognitive and mental organization (Dixon et al., 2012). Dynamical systems concepts as self-organized criticality or scale-free patterns have provided new insights about how the brain and the mind operate in a non-linear dynamic manner, self-organizing its activity always at the brink of criticality. The concept of self-organized criticality (SOC) (Jensen, 1998), one of the main exponents of this approach, was proposed by Bak et al. (1987) to define certain classes of dynamical systems which have a critical point as an attractor, displaying critical behavior without any significant “tuning” of the system from outside^1^. Critical systems present very interesting properties: the most notable of which is the lack of a dominant scale of activity. They show complex dynamical responses and their statistical properties have to be described by power laws. Thus, critical systems typically display temporal and spatial scale invariance in the form of fractals and 1/*f* noise, reflecting the process of propagation of long-range correlations based on local effects. The idea of long-range correlations refers to the presence of long-term dependencies in a signal between the current observation and a large set of previous observations, displaying a slow decay of the correlation function (typically exponential). Thus, the presence of long-range correlations suggests the presence of multiple, intertwined timescales in the system, responsible for the emergence of patterns or regularities in the system. For a multi-scale approach to cognitive science, SOC is appealing because it allows us to imagine systems that are able to self-regulate coordinated behaviors at different scales in a distributed manner and without a central controller.

1/*f* patterns have also been widely found in cognitive science and psychology. For example, 1/*f* noise is present in performance time series (Gilden, 2001). More recently, Van Orden et al. (2003, 2005) used 1/*f* noise measures in different tasks to gather evidence to argue that certain systems are not modular and decomposable but “softly assembled” systems sustained by *interaction-dominant dynamics* (IDD hereafter) as opposed to *component-dominant dynamics* (Van Orden et al., 2003). That is, IDD systems do not consist of additive interactions of their components, but multiplicative interactions that imply coordination between the different timescales in the system. Moreover, 1/*f* is not a unique and exclusive property of SOC or IDD systems (see Wagenmakers et al., 2004, 2012) since it has been shown to be displayed by a linear superposition of many random inputs with different time scales (Hausdorff and Peng, 1996). To avoid the uncertainty about the true origin of 1/*f* noise some authors have suggested to complementing it by a measure of multifractality as a quantitative indicator of the coordinated intermittency in the system's activity (Ihlen and Vereijken, 2010). Ihlen and Vereijken propose that intermittency is displayed within the series as distinct periods of large and irregular performance variability prompted by emergent changes in the commitment, attention to stimuli, or intention of the participant in a cognitive task. The multifractal spectrum width quantifies the difference between the intermittent and the laminar periods, so it provides further evidence of the interaction between different timescales in the system.

### 2.2. Outline

In this paper we try to explore the presence and relevance of multiple scale and inter-scale or long-range correlations in the perceptual crossing experiment. We think that genuine social interaction will display long-range correlations and coordinated intermittency in the form of 1/*f* scaling and a multifractal spectrum. Moreover, multi-scale interactions should be present in collective variables and not only in individual variables, as an indicator of an emergence of a social domain of interaction.

We propose a modified version of the original perceptual crossing experiment, in which the player only faces one opponent, which may be another human player or a programmed agent with two possible kinds of behavior (a simple oscillatory behavior or a “shadow” behavior that repeats the movement of the player. More information will be given in the next section and in the Supplementary Material Section S1). Thus, in our experimental setup we have different kinds of social interaction: humans recognizing each others as such, humans interacting with programmed agents with artificial behavior, humans failing to recognize other humans, bots tricking humans, etc. Can we characterize when genuine social interaction emerges? And if so, where does it lie?

In Auvray et al. (2009), the authors propose that the sensitivity for recognizing other intentional subjects, instead of being perceived by each of the participants, arises from the dynamics of the interaction itself. In their experiment, the distribution of clicks suggested that social recognition arose from a combination of (i) the ability to discriminate between mobile (human player, shadow) and immobile objects and (ii) the stability of mutual interaction patterns between two human partners or between human and a immobile object. This interpretation was inspired by the results in a simulated model which showed the importance of the stability of coordinated behavior (Di Paolo et al., 2008). However, we think that further evidence supporting the claim that social recognition emerges from interaction dynamics instead of individual sensitivity is necessary. In fact, the model presented in Di Paolo et al., (2008) could be interpreted as showing that relatively simple behaviors could account for a click distribution in which agents appear to “recognize” each other, without a genuine, underlying process of social recognition. We propose that genuine social interaction should arise from the emergence of a complex web of interactions across different timescales between the activity of different agents. For a first approach to support this claim we propose the following schema:

1. Since we consider that inter-scale dynamics might be relevant to characterize perceptual crossing dynamics, we perform measures similar to previous work in perceptual crossing experiments, and explore the existence of a link between our and previous results, and cross-scale interaction dynamics (Section 4.1).
2. We propose that if genuine social interaction is based on cross-scale interactions a fractal distribution should be present in collective variables of the social process. We propose the difference in the movement of the two players (using the difference between their speeds) as a candidate variable and perform fractal and multifractal analysis of the distribution in the individual rounds of the game, finding a clear 1/*f* and multifractal spectrum only when two human players interact (Section 4.2).
3. Finally, we suggest that as opposed to collective variables, the fractal structure of the individual dynamics of the player or their opponent alone should not be discriminative for the type of interaction going on. We analyze this issue repeating fractal and multifractal measures on the movement of the player and the movement of the opponent (using their individual speeds) and conducting linear mixed effects models to assess if the different variables analyzed (difference of speeds, speed of the player and speed of the opponent) can discriminate between the type of interaction, finding that only the collective variable of the relative speeds can discriminate the two types of programmed agents from genuine human interaction (Section 4.3).

## 3. Materials and methods

### 3.1. Experimental procedure

In this experiment, human participants were allocated computers to interact in pairs, within a shared perceptual space, where some opponents were other human participants and some opponents were computerized agents (bots) but participants were unaware of the nature of their opponents.

Our intention was not to make a duplication of Auvray's experiment where each participant simultaneously encounters a human partner, a mobile agent and a static one. In our case, each participant received only a single stimulus in one of the following scenarios: human vs. human, human vs. “oscillatory agent” and human vs. “shadow agent.” The “oscillatory agent” was programmed to deploy a sinusoidal behavior (describing a sinusoidal trajectory of 0.5 Hz and 200 pixels of amplitude), predictable and deterministic. In contrast, the “shadow agent” was able to show an irregular pattern because it consists of a “shadow image” of the participant (i.e., a bot that generates a movement strictly identical to the participant trajectory but delayed 400 ms. in time and 125 pixels in space). Participants were instructed to try to detect wether their opponent was human or not and asked to fill a questionary (although the analysis of the participants responses is out of the scope of this paper).

When participants arrived at the laboratory they were randomly assigned to a workstation and were provided with headphones. They were informed that the study involved two parts, each independent from the other and that the first one—training stage—would take approximately 3 min and the second one—evaluation stage—a further 10 min. In order to guarantee confidentiality during the study, identification codes/nicknames were chosen by the participants. Throughout the experiment, participants were provided with verbal instructions regarding the structure of the experiment and their sections.

In the training stage, the participants were informed that it was a simple “proof of concept” stage and that the purpose was only to learn how the platform worked. Participants were free to move the mouse as they pleased during three sessions of 1 min each with a short break between them. They played consecutively against three bots of increasing difficulty in the interaction: a static bot, a bot moving at a constant low speed and a bot moving at a constant medium speed.

After that, they were informed of the aim and rules of the evaluation part of the experiment. The experiment consisted of 10 sessions of 40 s each. In each session: (i) each participant was randomly assigned an opponent (human-human or human-bot) to explore the virtual space; (ii) participants were asked to move their mouses in order to detect the movement of their assigned opponents, (iii) after each session, participants were asked to make a choice between the two options displayed on the screen in order to guess whether their opponent was a human or a bot, and (iv), finally, participants were informed on the screen whether or not they had guessed successfully. After the 10 sessions were completed, the experiment was declared finished.

A total of 13 participants (8 females and 5 males) took part in this experiment. Their ages ranged from 16 to 19 years. However, due to a problem with the computer of one participant, some data were not recorded and therefore not included in the study. As well, we removed a few samples in which no interaction between the players was detected. The final dataset used in the analysis comprises a total of 106 samples of the cursor positions over time of each participant recorded with a sampling period of 1 ms.

More detailed information related to experiment protocols (study sample, characteristics of participants, experimental stages, number of sessions, etc.), information about how the technological platform was built (network properties, latency estimation, etc.) or how software requirements were programmed (virtual environment conditions, experimental devices, sensor stimuli, etc.) can be consulted in the Supplementary Material Section S1.

### 3.2. Fractal and multifractal analysis

In order to analyze the interaction between the subjects, we take the time series of the distance between the two players (or the player and the bot agent). We compute the players relative velocity (i.e., the first derivative of the distance between the player and its opponent) to extract whether the players are approaching or distancing themselves at each moment of time. Then we use a DFA algorithm (Peng et al., 2000) to compute the statistical self-affinity in the data series of distance variations and, in order to verify if the involved cognitive processes presents an intermittent non-linear structure, we also analyze the multifractal spectrum with the multifractal DFA (MFDFA) algorithm (Ihlen and Vereijken, 2010).

In a nutshell, the DFA algorithm removes the mean and integrates (cumulatively sums) the analyzed time series *x*(*i*) into *y*(*k*) and then divides it into segments of equal length *n* (i.e., of a certain time scale). For each segment, a least squares line (the trend of the signal within that segment) is fitted to the data obtaining a local linear approximation *y*_*n*_(*n*). The characteristic size of the fluctuation *F*(*n*) is computed as the root mean square deviation between the integrated signal and its trend in each segment. This computation is repeated for every value of *n*.

(1) $$y(k)=\sum_{i\;=\;1}^{k}x(i)$$

(2) $$F(n)=\sqrt{\frac{1}{N}\sum_{k\;=\;1}^{N}{\left[y(k)-y_{n}(k)\right]}^{2}}$$

where *N* is the total length of *x*(*n*). Typically, *F*(*n*) increases with *n*. A linear relationship on a log-log plot with slope α indicates the presence of fractal scaling in the analyzed signal, where α is a generalization of the Hurst exponent, and is related to the scaling in the Power Spectrum of the Fourier analysis being β = 2·α − 1.

The DFA has some advantages compared to spectral analysis as the Fourier transform. While the Fourier transform is only well suited for stationary signals, the DFA has been reliably used in non-stationary signals (Kantelhardt, 2011). A visual inspection of the data revealed abrupt transitions at different moments, so we decided to use DFA instead of the Fourier transform. Usually pink or 1/*f* noise is considered to correspond to values of β between 0.5 and 1.5. Similarly, values of β close to 0 correspond to white noise (uncorrelated processes) and values close to 2 to brown noise (process driven by slow timescales showing short-term predictability). Only processes with β around 1 and a wide multifractal spectrum are considered to display SOC (Jensen, 1998; Ihlen and Vereijken, 2010).

On the other hand, the multifractal spectrum is computed by the MFDFA algorithm, a variation of DFA in which the squared exponent of the root mean squares deviation becomes a variable *q*, therefore allowing calculations outwith the standard euclidean norm defined by the root mean square. Following this procedure, positive *q*-values describe the scaling behavior of the segments with large variance because the large deviations from the corresponding fits will dominate the average *F*(*n*). On the contrary, negative *q*-values describe the scaling behavior of the segments with small variance because the large deviations from the corresponding fits will be largely attenuated on the average *F*(*n*) (Kantelhardt, 2011). This behavior describes the regularity of laminar periods of little performance variability vs. the regularity of intermittent periods of large performance variability, and can be quantified as the difference between the maximum and minimum values obtained along the different *q*-values, namely the width of the multifractal spectrum. A multifractal signal is characterized by the presence of intermittent periods of large and irregular fluctuations, denoting the interaction among time-scales within the signal. Being the width of the multifractal spectrum, a measure of these interment periods, it serves as an index to quantify an structure of interactions between temporal scales (Ihlen and Vereijken, 2010).

DFA bins for parameter *n* have been defined logarithmically from 2^6^ s to $\frac{1}{4}$ times the size of the time series and an intervals of 2^0.01^ s. For the MFDFA we have used the same values for the *n* bins and we have taken a value of *q* with values from −3 to 3 with intervals of 0.25.

### 3.3. Statistical approach

The design of this experiment involves repeated measures per subject and, in order to account for this characteristic, linear mixed effect models have been computed. In a nutshell, mixed effect models are regression models that incorporate both fixed and random effects. Fixed effects are the independent variables of interest while random effects replicate the structure of the data (i.e., games within player in this case). As a consequence, the unexplained variation can be split into the variation between players and the residual variation between games within players. In this experimental design, the variable “type of opponent” (“human,” “shadow agent,” or “oscillatory agent”) acts as the only fixed effect. Each player performs the experiment several times, so we include the variable “player” in order to account for the potential lack of independence of the repeated measures for each participant. In the next section, these techniques will be applied to the results of the experiment, showing the statistical validity of our study. More detailed description of the method can be found in the Supplementary Material Section S2.

## 4. Results

Above we proposed that some previous analysis made about the scale in which the dynamics of the perceptual crossing should be considered. We proposed instead that multi-scale analysis is better suited to unveil the structure of social interaction. In this section we perform different tests to explore the possibility of multi-scale interactions shaping the dynamics within the perceptual crossing experiment. We start by analyzing our results with measures similar to some used in previous analysis and propose the necessity of complementing them with other measures that are not constrained to one particular scale of behavior.

### 4.1. Preliminary analysis

Typically, analysis of the interaction dynamics in the perceptual crossing has not been concerned with the distribution of activity across different scales. For example, in Auvray et al. (2009) the two variables that explain the detection of another human player are the frequency of stimulation (the number of times a player receives an input from its opponent) and the probability of clicking (the probability of the player clicking their mouse in a 2 s interval after a stimulation). The setup in our task differs in that the players are not asked to click if they recognize a human player. However, here we substitute the probability of clicking for the probability of having a new stimulation in an interval defined as a given window after a previous stimulation. This measure is intended to capture the probability of engagement in an ongoing interaction between the two players. Unlike Auvray et al. (2009) we will not use a single value for the window length and will instead test the values 0.25, 0.5, 1, and 2 s (around 95% of stimulations happen within a window of 2 s after the previous stimulation). We will refer to the frequency of stimulation as *F*_*s*_ and the probability of consecutive stimulations in a window of length *L* seconds as *P*^*L*^_*s*_.

We conduct linear mixed effects modeling of the series corresponding to each measure and we obtain the results shown in Table 1. Here we show the *p*-value resulting from the comparison of the distributions corresponding to players when playing against an other human player and when playing against each type of bot. We can observe in the table how the frequency of stimulation *F*_*s*_ does not discriminate against different types of players. This result is different from the classical perceptual crossing results, and maybe caused by the fact that the participants play individually against each type of opponent. For the probability of consecutive stimulations *P*^*L*^_*s*_ we observe that the result depends largely on the chosen value of *L*. For example, for the extreme values of 0.25 and 2 s we cannot discriminate human opponents against either of the two bots (setting the statistical significance level at 5%). Oscillator opponents however can be discriminated for windows of 0.5 and 1 s, and shadow opponents can only be discriminated for windows of 0.5 s. Thus, choosing a value of 0.5 s would give us a variable that allows us to statistically differentiate the different players, showing us that at that particular scale some opponents have more consecutive stimulations with the player than others (in this case, the shadow agent presents a higher probability of consecutive stimulations).

**Table 1:** **Results of the linear mixed-model effects for comparing stimulation frequency *F*_*s*_ and probability of consecutive stimulations *P*^*L*^_*s*_ between the rounds where the player was facing other human player and the two cases of programmed agents (oscillatory and shadow agents)**.

**Groups**	*****p***-value**				
	***F*_*s*_**	***P*^0.25^_*s*_**	***P*^0.5^_*s*_**	***P*^1^_*s*_**	***P*^2^_*s*_**
human-human vs. human-oscillatory	0.2381	0.0000	0.0000	0.0000	0.0496
human-human vs. human-shadow	0.6591	0.6159	0.0000	0.2455	0.0519

**Figure 1:**
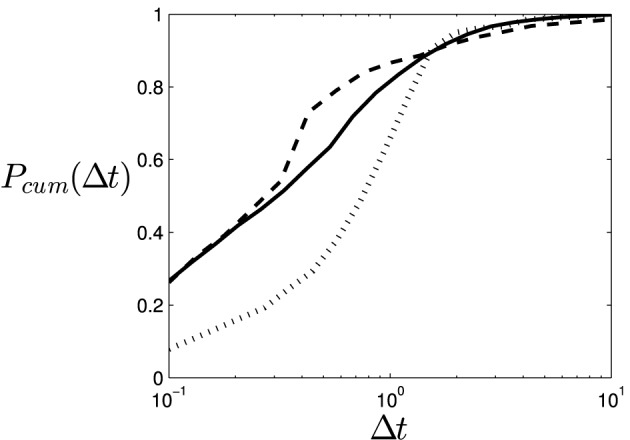
**Cumulative probability density function of the time between collisions for different types of opponents aggregated among participants and trials**. Values for the regions illustrated are: (dotted line) human vs. oscillatory agent, (dashed line) human vs. shadow agent, (solid line) both participants are human players.

To asses the significance of the statistical results without the bias of choosing particular windows of analysis, we proceed to compute the distribution of inter-stimulation intervals Δ*t*, that is, the time between one stimulation and the next. However, since the data for each player and round are scarce (around 40 mean stimulations by game, depending on the type of agent), we aggregate the data of different players and rounds (although this could entail losing some information about the data structure). The result of the cumulative probability can be observed in Figure 1, where we observe that the windows of discrimination in Table 1 roughly coincide with the intervals in which the cumulative density functions overlap. This may indicate that what we are doing when we just take the probability of stimulation (or clicking) is integrating the density distribution of a process that unfolds over different scales (in our case ranging from 0.1 to 10 s).

**Figure 2:**
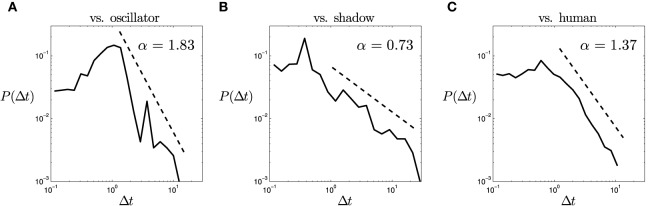
**Probability density function of the time between stimulations for different types of opponents aggregated among participants and trials**. Values for the regions illustrated are: **(A)** human vs. oscillatory agent (“vs. oscillator”), **(B)** human vs. shadow agent (“vs. shadow”), **(C)** both participants are human players (“vs. human”).

Here we may question whether the fact that the results for a particular window are discriminative between agents is either the consequence of something relevant happening at that timescale, or it is instead caused by the different underlying structures of the temporal density distributions. In order to shed some light on this question we have represented the aggregated density distribution functions of the time between stimulations Δ*t* for the three types of opponents (Figure 2). In the figure we can observe the presence of long tails that start around 0.5 s in the case of the shadow and human opponents, and that these long tails have different slopes in a logarithmic plot. This might be indicating that the statistically significant differences in the activity between the different 0.5 s windows are not the result of something happening at that scale, but the product of a deeper change in the temporal structure of the interaction. In that case the statistical difference at windows of 0.5 s may appear because the fact that we are integrating along all the smaller timescales.

**Figure 3:**
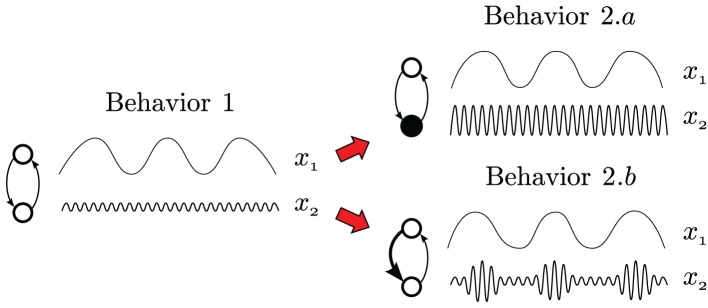
**Example of statistical comparison between two multiscale systems**. In case 2.a the behavior is statistically different from 1 at scale *s*_2_ because the intrinsic levels of activity at this scale have been increased. However, in case 2.b the statistical differences respect to 1 at scale *x*_2_ is not due to any intrinsic change in *x*_2_ but instead to a change in the relation between *x*_2_ and *x*_1_, that now presents a phase modulation from slower to fastest frequencies.

To illustrate this point we offer the following example (Figure 3): imagine that we have a system in which we can access to two components *x*_1_ and *x*_2_, each one being active at a different timescale. The same system may display different behaviors. We can imagine that stimulating *x*_2_ the system switches from behavior 1 to behavior 2.a. As a result of the behavior change, we can find statistical differences between the distributions of *x*_2_ in behavior 1 and 2.a, respectively. Also, we can consider a different condition in which we enhance the influence of variable *x*_1_ over *x*_2_ (in a process of phase modulation), making the system switch from behavior 1 to behavior 2.b. Again, we find statistical differences between the distributions of *x*_2_ in behavior 1 and 2.b. The important point is that, while in the first case the statistical distribution of *x*_2_ is provoked by a direct change in the activity of this variable (directly stimulating the component that produces it), in the second case the statistical difference in *x*_2_ can only be explained by a change in the interaction between variables *x*_1_ and *x*_2_. Similarly, significant statistical changes in a timescale of 0.5 s, might be the result of something relevant happening at that scale, or it may be the result of a reconfiguration of the whole temporal structure relating different scales of behavior.

The example in Figure 3 indicates that by analyzing just one particular scale of the system we may be failing to capture the causes of a change in the system's behavior even in the case that we were able to find a statistical discrimination of the distribution of a variable. In the case of the perceptual crossing, we propose that previous analysis may be extended with analysis of the activity at different scales and the relation between these scales. We contend that taking into account the changes in the temporal structure of inter-stimulation times allows a fuller explanation of the statistical discrimination offered by simple indices such as the number of clicks or consecutive stimulations within a given window. Nevertheless, the analysis of the density distribution of aggregated data is too coarse to test this claim. We need to perform a more detailed analysis of the temporal structure within the individual interaction dynamics in each round to provide more conclusive results. We propose that statistical analysis of fractal and multifractal time series may be a better suited tool for this kind of problem.

### 4.2. Fractal dynamics in the interaction process

In this section we seek a more detailed analysis of the temporal structure of the interaction between the two players for the three kinds of opponent. In doing so, we need to extract the movements of the two players. In order to analyze the interaction between the subjects, we take the time series of the distance between the two players (or the player and the bot agent): (i) the first derivative of the distance is computed in order to obtain the variations in the distance (whether the players are approaching or distancing themselves at each moment of time given that we are interested in the coordinated movements of the players, not their positions); (ii) we use the DFA and MDDFA algorithms to compute the structure of correlations across scales in the data series and (iii) we perform a linear mixed-effects modeling in order to observe if the DFA and MDFA exponents are capable of differentiating between the interaction dynamics depending on the type of opponent the player is facing (oscillatory, shadow or human).

**Figure 4:**
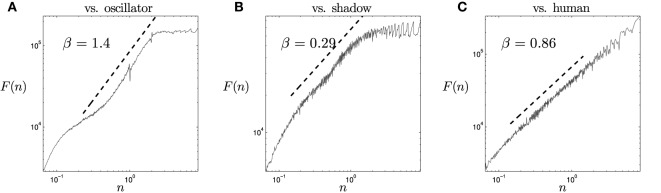
**Fractal analysis calculated on interactive patterns between two participants**. Values for the regions illustrated are: **(A)** human vs. oscillatory agent (“vs. oscillator”), **(B)** human vs. shadow agent (“vs. shadow”), **(C)** both participants are human players (“vs. human”). The examples are representative cases of the three kinds of populations in the experiment.

As a first step in the analysis, we observe the results of individual DFAs in different rounds. In Figure 4 we show some representative examples of the types of temporal structures displayed by the interactions with each type of agents. Since the slope of the fluctuations in a logarithmic plot is not always linear for all scales, we check if there is any cutoff value in which the linear relationship is truncated. We do this by searching for negative peaks in the second derivate of *F*(*n*). The search of cutoff values is only performed in the right half of the *n* axis, in order to find only the cutoffs at larger scales. Once the cutoff value is found, we analyze the slope *F*(*n*) for the values of *n* in the decade just below the cutoff value (e.g., Figures 4A,B). In the cases where there is no cutoff value (as in Figure 4C) we analyze the interval *n* ∈ [10^−0.5^, 10^0.5^].

For the oscillatory agent, we can observe in Figure 4A a flatland at higher values of *n*, followed by a steep linear slope with a β parameter around 1.5. For lower values of *n* the linear slope disappears. This kind of fluctuation is characteristic of oscillatory dynamics, with the transition from flat to slope being equal to the period of the oscillations. In the other case, for the shadow agent, Figure 4B presents something similar to a linear slope in the middle of the fluctuation spectrum, although the slope linearity breaks at the extremes. The slope of the fluctuation gives an exponent somewhat higher than β = 0. This suggests that weak short-range correlations exist (close to a white noise structure), but they do not hold for longer timescales. Finally, in Figure 4C, when a player faces another human player, the fluctuation spectrum displays a linear slope with a β exponent close to a pink noise spectrum (β = 1). In a large part of the series, the fractal slope reaches the largest timescales, showing that the correlations of the interaction dynamics cover a wide range of the spectrum. In Figure 4C, fractal relations covering the hole spectrum are illustrated, although there are many other cases which present a cut-off point at large scales breaking the linear relation. We propose that the existence of fractal 1/*f* patterns covering the whole analyzed spectrum just in some cases of human-human interaction may be related with the fact that in some cases interaction will be successful during the some round but other cases will experience a breakdown in the interaction, leading to disruption in correlation at longer timescales.

**Figure 5:**
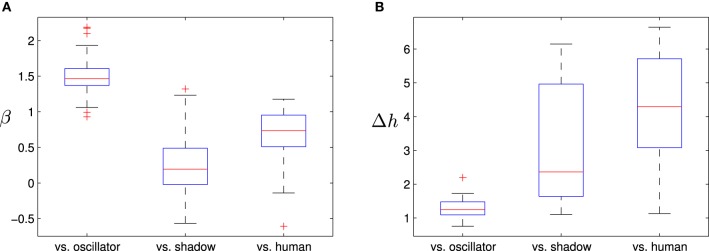
**(A)** Boxplots distribution of β and, **(B)** width of the multifractal spectrum Δ*h* in the time series of the relative velocity between participants. Values illustrated refer to interactions between: a human and a oscillatory agent (“vs. oscillator”), a human and a shadow agent (“vs. shadow”) and two human participants (“vs. human”).

Figure 5 shows three particularly representative examples of the three kinds of populations in the experiment. Particularly, in Figure 5A we can observe the boxplots of β for the different types of interaction. When the opponent is the oscillatory agent, we find that the values of β in the time series are around 1.5. This means that the interactions are closer to a brown noise structure, signifying that the interaction is more rigid and structured than in the other cases. This makes sense since the movement of the oscillatory agent is constraining the interactions into its cyclic movement structure. On the other hand, when the opponent is the shadow agent, we have the opposite situation in which the interaction dynamics tend to display values of β greater but close to 0. This means that the history of interaction is more uncorrelated. Using a linear mixed-effects model we asses that indeed the three distributions of β are different from each other. We tested this idea appropriately using linear mixed-effects models of the three types of opponents (oscillatory agent, shadow agent, and human) to assess the presence of statistically significant differences between the density distributions of β. Using a linear mixed-effects model we can test that beta is a significant parameter for distinguishing the different kinds of interactions depending on the type of opponent [*F*_(2, 93)_ = 258.350, *p* < 0.0001].

As we have mentioned above, fractal analysis is a mathematical procedure to determine scale invariant structures in a dataset. Monofractal signals have the same scaling properties throughout the entire signal, therefore, can be indexed by a single global exponent (that is known as the Hurst exponent, see Section 3.2). Alternatively, when spatial and temporal variations in a scale invariant structure appear, we get a “multifractal structure” that can be decomposed into different subsets characterized by different local Hurst exponents (denoted as *h*) which quantify the local scaling of the time series. With this collection of exponents, we characterize their scaling properties: any deviation from the average fractal structure for segments with large and small fluctuations is captured by the “multifractal spectrum width,” denoted by *D*(*h*). In particular, the resulting multifractal spectrum is represented by an arc defined as the difference between the maximum and minimum values of the local Hurst exponent for each scale [*D*(*h*) vs. *h*]. Thus, the width of this spectrum is a measure of the degree of multifractality and will be zero for a monofractal series. The higher the value of the width the more multifractal the spectrum will be.

In order to verify the non-linear intermittent structure of the involved processes behind the patterns analyzed above, we also analyze the width of the multifractal spectrum of the derivative of the distance between players. For each case, we calculate the width of the multifractal spectrum using the MFDFA algorithm and plot the distributions of the obtained values depending on the type of opponent (Figure 5B). The probability distribution of the multifractal spectrum width Δ*h* on the oscillatory agent is more concentrated around small widths, indicating little interaction between the time-scale of the oscillation frequency of the agent and the time-scales of the movement of its human opponent. Larger values on the distribution of the shadow agent indicate stronger interaction between its time-scales. Finally, the distribution of the human agent reaches the largest values of the multifractal spectrum width, suggesting a rich time-scale dynamics prompted by the interactivity between the time-scales of the movements of a pair of human opponents. Again, a linear mixed-effects models shows us that the distributions of values of Δ*h* are different depending on the type of opponent [*F*_(2, 93)_ = 258.350, *p* < 0.0001].

The fractal and multifractal spectrum results show that the relative velocity of the player with respect to their opponent in the interaction process present different distributions depending if genuine social interaction is happening or the player is interacting with an artificial agent with trivial (oscillatory) or complex (shadow) patterns of movement. It is interesting that 1/*f* noise emerges for a collective variable (the derivative of the distance) only in the case of human-human interaction, suggesting that long-range correlations emerge in the shared space of social interactions and genuine social interaction is characterized by the collective evolution of the dyadic exchange. In those cases where the interaction between the players is too rigid or too weak, the emergent multiscale phenomenon disappears. Multifractal seems to support this claim. To further test this proposal and determine if the same results can be obtained from non-collective variables, we will compare this results with the behavior of individual variables of the player and their opponents.

### 4.3. Comparing fractal exponents in individual and collective variables

One of the ideas behind much of the work in the perceptual crossing paradigm is that the interaction between subjects is a constitutive element of social cognition (Auvray et al., 2009). If that is true, the characteristics of a genuine social interaction should appear only in dyadic variables such as the relative velocity between subjects and should be absent in individual variables such as the individual movement of the player or their opponent. For testing to what extent this is true, we repeat the fractal and multifractal analysis above using the velocity of the player and the velocity of their opponent, instead of the relative velocity between the two. Thus, we can test if the differences in the fractal emergent structure takes place in the shared space of social interaction or are instead phenomena that may be accounted for by the changes in individual dynamics alone.

**Figure 6:**
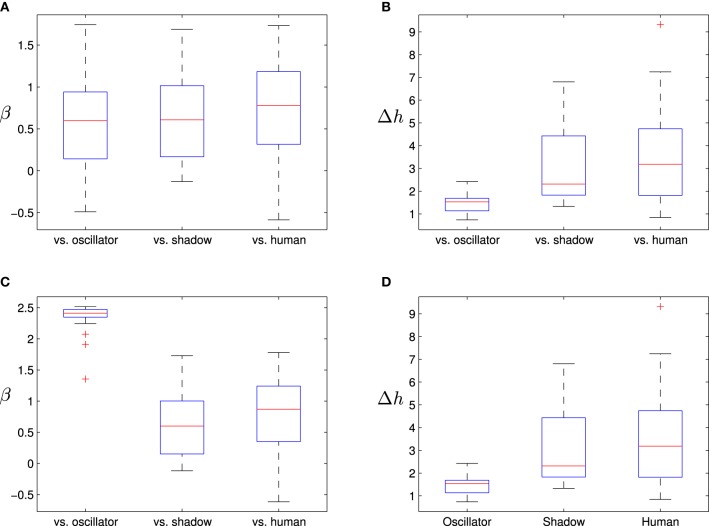
**Boxplots distribution of β (left side) and width of the multifractal spectrum (right side) in the velocity of the players**. The upper figures **(A,B)** represent the fractal and multifractal analysis when we take the velocity of the player. The bottom figures **(C,D)** represent the case when we analyze the velocity of the opponent. Values illustrated refer to interactions between: a human and a oscillatory agent (“vs. oscillator”), a human and a shadow agent (“vs. shadow”) and two human participants (“vs. human”).

In Figure 6 we can see how in this case the boxplots of β and the multifractal spectrum width Δ*h* show more overlapping among the distributions corresponding to the different opponents.

We tested this proposal appropriately using linear mixed-effects models of the three types of opponents (oscillatory agent, shadow agent and human) to assess the presence of statistically significant differences between the density distributions of β (Table 2) and Δ*h* (Table 3) for three different cases: (i) the relative velocity between the player and its opponent (labeled in the tables as the “interaction” case), (ii) the individual velocity of the player (labeled as “player”) and (iii) the individual velocity of the opponent (labeled as “opponent”). Both tables include the corresponding *p*-values resulting from the modeling.

**Table 2:** **Results of the linear mixed-model effects for comparing the fractal β exponent from DFA results between the rounds where the player was facing other human player and the two cases of programmed agents (oscillatory and shadow agents)**.

**Groups**	***p*-value**		
	**Interaction**	**Player**	**Opponent**
human-human vs. human-oscillatory	0.0000	0.1106	0.0000
human-human vs. human-shadow	0.0017	0.6831	0.0850

*The left column (interaction) reflects the results when the relative velocity between the players is analyzed, central column (player) shows the results when the velocity of the player is analyzed and the right column (opponent) the velocity the opponent*.

**Table 3:** **Results of the linear mixed-model effects for comparing the fractal Δ*h* exponents from MFDFA results between the rounds where the player was facing other human player and the two cases of programmed agents (oscillatory and shadow agents)**.

**Groups**	***p*-value**		
	**Interaction**	**Player**	**Opponent**
human-human vs. human-oscillatory	0.0000	0.1405	0.0000
human-human vs. human-shadow	0.0002	0.8594	0.4601

*The left column (interaction) reflects the results when the relative velocity between the players is analyzed. Central column (player) shows the results when the velocity of the player is analyzed and right column (opponent) the velocity of the opponent*.

Given the results shown in both tables and setting the significance level at 5%, we can conclude that only in the case of the relative velocity between the agents (“interaction” columns) all three distributions are statistically significantly different for both β and Δ*h*.

For the case of the velocity of the player, we cannot assure an statistically significant difference between the distributions of β and Δ*h*. In the case of the velocity of the opponent, we could only find evidence of statistically significant differences between the oscillatory agent and the other two kinds of opponents, but not between the human opponent and the shadow agent.

The obtained results show that individual variables are not suitable for discriminating between the kind of interaction going on in the case of the shadow agent. This reveals that when the individual behaviors have some kind of complexity, what it is relevant in terms of the emergence of social interaction is what is going on in the interaction between the two subjects and not the complexity of their individual behaviors.

## 5. Discussion

In this paper we have revisited some of the results of the research program around the perceptual crossing paradigm. As we have seen, in recent years, this paradigm has allowed the study of social interaction in its simpler form and has offered very interesting experimental results to try to understand what kind of processes underly the emergence of social engagement. In particular, we have addressed a new version of the experiment in which the player can face only one human player or an artificial agent that shows either (i) an oscillatory movement or (ii) behaves as a temporal “shadow” of the player. After analyzing the different kinds of social engagement dynamics generated, we have found that a fractal 1/*f* structure (with high multifractal indices) at many timescales of the history of collective interactions only emerges in the case of genuine social interaction (i.e., the “human vs. human” case) and not in other cases (“human vs. agent”). In this respect, our results present a new interpretation of the results obtained in previous perceptual crossing experiments: there could be some limitations in the approach take in previous analyses of the social engagement process, which have been often restricted to studying a single temporal scale and consequently falling short for capturing the complex unfolding of the different levels of cognitive and social interaction.

This interpretation offers a new conceptualization of the directions in which we should focus attention: given the results shown in this paper, it is possible that the emergence of social engagement might not depend solely on either the stability of co-regulative dynamics between two participants as suggested in previous perceptual crossing experiments and simulations (Di Paolo et al., 2008; Auvray et al., 2009). Furthermore, the results obtained let us propose that genuine social engagement might be better characterized by a structure of cross-scale interactions that we try to capture analyzing fractal 1/*f* scaling and multifractal spectrum. Moreover, fractal and multifractal exponents showed no statistically significant differences when we analyzed the velocity of the player or their opponent compared to the relative velocity between them, leading us to conclude that the emergence of a 1/*f* structure for genuine social interaction is something that happens only in the shared space between the two subjects, and the process cannot be reduced to the individual dynamics of any of them.

However, this work leaves several questions unanswered. The first concerns what an adequate framework of analysis might be and how previous and new insights can be integrated in a larger framework. The framework presented here still needs to be extended, since 1/*f* scaling and the multifractal spectrum reduce the complexity of multiscale dynamics to a single exponent that, although detecting the presence of activity at different scales, falls short of being able to characterize the nature of cross-scale interactions in detail. Multi-scale synchronization analysis employed for measuring inter-brain synchronization in social tasks appears to be a suitable candidate for extending the analysis presented here with multiscale synchronization analysis of behavioral dynamics (Dumas et al., 2010; Müller et al., 2013). More detailed analysis may also offer new points of connection with previous work and alternative explanations for the phenomena observed in the perceptual crossing experiment.

Another way forward may lie in modifications of the perceptual crossing experiment which may prove helpful in better understanding the cross-scale interactions in minimal social interaction. Interesting advances following this approach include the work of Iizuka et al. (2013), which studies the emergence of a communication system between two participants, using the perceptual crossing set up to collectively categorize different symbols. Also, (Froese et al., 2014b) explore the evolution of interaction of fixed pairs of players during several rounds with the objective of establishing a team for finding each other, observing that at some point the players simultaneously become aware of each other. This kind of extended experiment may allow the study of correlations at larger scales than just instantaneous online recognition, allowing us to analyze interesting dynamics as learning, development of shared patterns and joint development of the player's mutual dynamical entanglement.

Finally, it could also be interesting to apply some of these ideas to the simulation domain. Some of the attempts to model agents that could perform the perceptual crossing task were based in an agent vs. agent joint evolution using a genetic algorithm maximizing the number of interactions between the agents. We are concerned that such minimalistic scenarios as the perceptual crossing experiment may bias co-evolution of agent toward simple behaviors that exploit only one scale of behavior to maximize the outcome (e.g., simple oscillatory behavior). Maybe other evolution strategies could be used, for example trying to evolve turn taking behavior (Iizuka and Ikegami, 2004). Another interesting extension to tackle this problem could be to explore the possibilities of mixed environments shared by human and robotic agents in order to allow a richer repertoire of dynamics that could be exploited for learning and tuning of the modeled agents.

### Conflict of interest statement

The authors declare that the research was conducted in the absence of any commercial or financial relationships that could be construed as a potential conflict of interest.

## Footnotes

^1^All the data used in this experiment is available at https://github.com/IsaacLab/datasets/tree/master/PerceptualCrossing/data-28-03-2014
