# Supplementary material for: Quantifying long-range correlations and 1/f patterns in a minimal experiment of social interaction
Source: Front Psychol. 2014 Nov 12;5:1281. doi: 10.3389/fpsyg.2014.01281 (PMC4228835; doi:10.3389/fpsyg.2014.01281)
Supplement: Supplementary file 1 [file Presentation1.PDF]

Frontiers in Cognitive Science

Supplementary Material: Quantifying long-range correlations and $1/f$ patterns in a minimal experiment of social interaction

Manual G. Bedia [*], **Miguel Aguilera, Tomas Gomez, David G. Larrode, Francisco Seron**

Correspondence*:
Manuel G. Bedia
Dept. of Computer Science, School of Engineering and Architecture (EINA), University of Zaragoza, Maria de Luna s/n, 50018 Zaragoza, Spain, E-mail: mgbedia@unizar.es

Towards an embodied science of intersubjectivity: Widening the scope of social understanding research

1 EXPERIMENTAL PROTOCOLS: PARTICIPANTS

Thirteen participants (8 females and 5 males) took part in this experiment. Their ages ranged from 16 to 19 years. Calculations were performed from all variables on all participants with one exception: data for one participant were incomplete and not recorded due to a problem with her computer. Before the experiment, participants were instructed on how the experimental protocol worked, and what the rules of the game were. They gave their informed consent to the experimental procedure. The study was approved by the Clinical Research Ethics Committee of Aragon (CEICA) from the Aragon Health Sciences Institute (IACS). The experiments took place in a virtual environment, built purposefully with a software tool for test-bed experiments of social interaction.

1.1 EXPERIMENTAL PLATFORM

The platform of the experiment consisted of a local computer network connecting a server to work-stations for the participants. The advantage of this infrastructure is its reproducibility and scalability potential, which was proven by the fact that only standard computer equipment was required to perform some of the extensive experimental possibilities offered.

Briefly, the server (ApacheTomcat©) runs the management tools, which consist of a web-based server application for the configuration of the perceptual crossing parameters, plus a client-server application to establish a bi-directional communication with the participants. The implemented technology (webSockets) allows sending messages with little overhead, therefore resulting in a very low latency connection essential for our experimental conditions. The local network was built with a 6-port switch working on 10 Mbps and the participants connected to the server with a standard web browser (Mozilla Firefox©). The client-server application is the counterpart of the communication with the server previously described. The front-end software framework, programmed in Java and running on a standard web-browser, created the perceptual space with which participants engaged. The movement of the participants within this space was controlled with a computer mouse. During the set up of the experiment, we made some tests to provide an estimation of the communication performance. In order to determine the range of latency of the system, a Network Protocol Analyzer (wireShark©) was used to

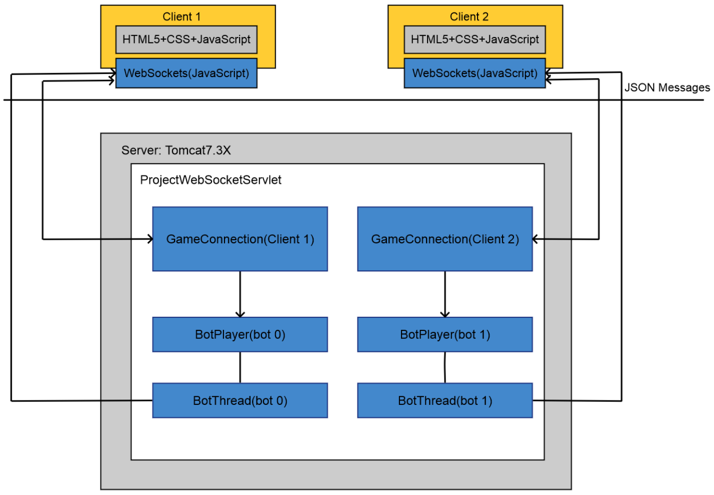

Supplementary Figure 1. Architecture diagram showing the experimental platform

capture the packets transmitted and received via a Network Interface Card. The results for communication latency estimation for ten consecutive request/response couples, with some milliseconds interval between them, had a maximum latency of $40 - 50$ ms. This means that, through the platform, the action performed by one participant on her computer reaches the computer of her partner with a maximum delay of 50 ms. This performance is in line with the those of other experiments on interaction.

1.2 SOFTWARE APPLICATION AND DEVICES

The environment consisted of a virtual one-dimensional space 800 pixels long with both ends connected, forming a torus to avoid the singularities induced by the edges. The input device used by the participants consisted of a computer-mouse that moved from left to right searching for someone with whom to interact.

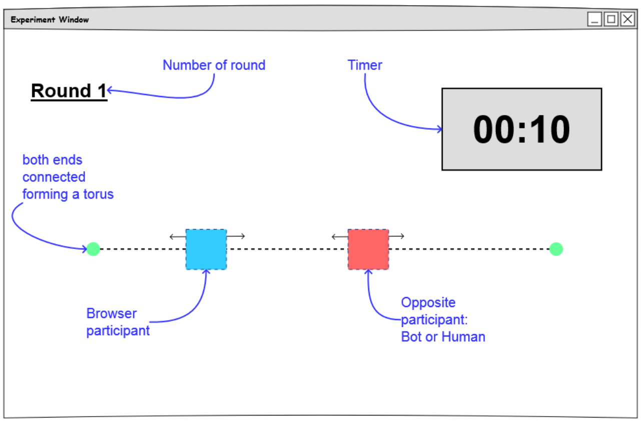

Supplementary Figure 2. Schematic illustration of the basic parts in the software environment.

When the participant moved her computer-mouse, the cursor in this space shifted. When the cursor of a subject crosses the cursor of the other participant a human or an artificial agent- the collision is

perceived. In particular, the sensorial stimuli prompted during the experiments were reduced to simple audible signals. Although it was possible to provide audible, visual or a combination of both stimuli, the experiments were organized simply using an audible signal. When opponents (either other human or a bot) cross their cursors, they receive an audible stimulus lasting 500 ms. No image of the cursors or their positions were displayed on the computer screen, so the audible stimuli were the only environmental perceptions of the virtual space. The sequence of movements executed by the participants and the corresponding sensory stimuli they received were recorded in each experimental session. The average success rate for the whole group was 55.43%.

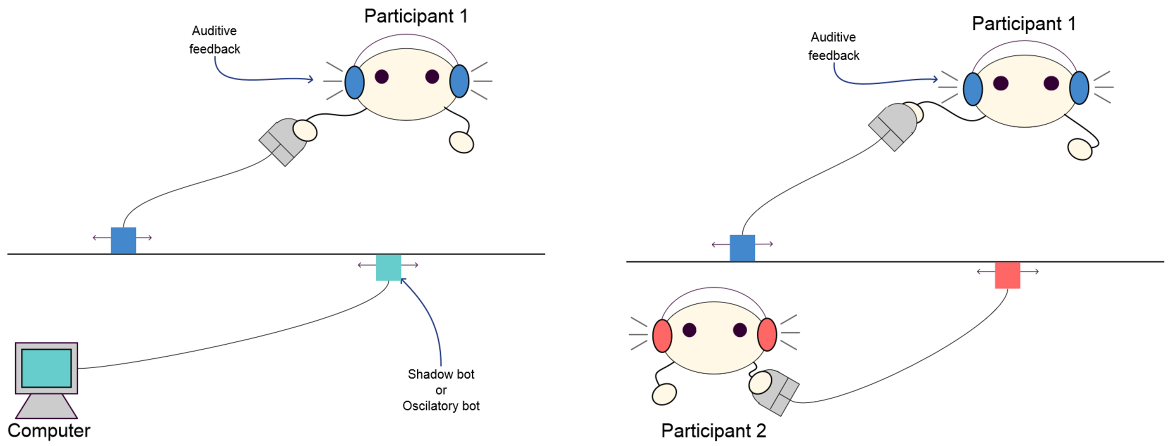

Supplementary Figure 3. Schematic illustration of the experimental set-up. (Left side) A 'human-software agent' interaction is represented. (Right side). The figure represents two human players trying to recognize each other.

1.3 DATA COLLECTION AND MODEL ANALYSIS

During every experimental session, the position described by each participants cursor was recorded in a file which also stored a timestamp attribute. All the data used in this experiment is available at: https://github.com/IsaacLab/datasets/tree/master/PerceptualCrossing/data-28-03-2014.

From these data, changes in the distance between participants in every session were calculated generating only one time series for every session which have been the focus of further analyses.

In order to quantify the fractal-like autocorrelation properties of each of the time series generated, a detrended fluctuation analysis (DFA) algorithm was used that estimated the scaling exponent in every fractal series, characterizing long memory dependence phemomena. As we did not assume that the processes were normally distributed, calculations were completed through multifractal detrended fluctuation analysis (MFDFA). The DFA and MFDFA code used in this paper is part of MatLab Toolboxes©: code for DFA can be downloaded from http://www.nbtwiki.ne and MFDFA code is available at http://www.ntnu.edu/inm/geri/software.

Analysis of the fractal study data included descriptive statistics and mixed effects models of the relationships within the new measurements. These models allowed us to account for the variation between players and the residual variation between the different games played by each respondent. Models were developed using the nlme package in the open-source statistical software R (http://cran.r-project.org/web/packages/nlme/index.html).

1.4 SOFTWARE AGENTS: OSCILLATORY AGENT AND SHADOW AGENT

In the experiments, each participant received only a single stimulus in one of the following scenarios: human vs. human, human vs. 'oscillatory agent' and human vs. 'shadow agent'.

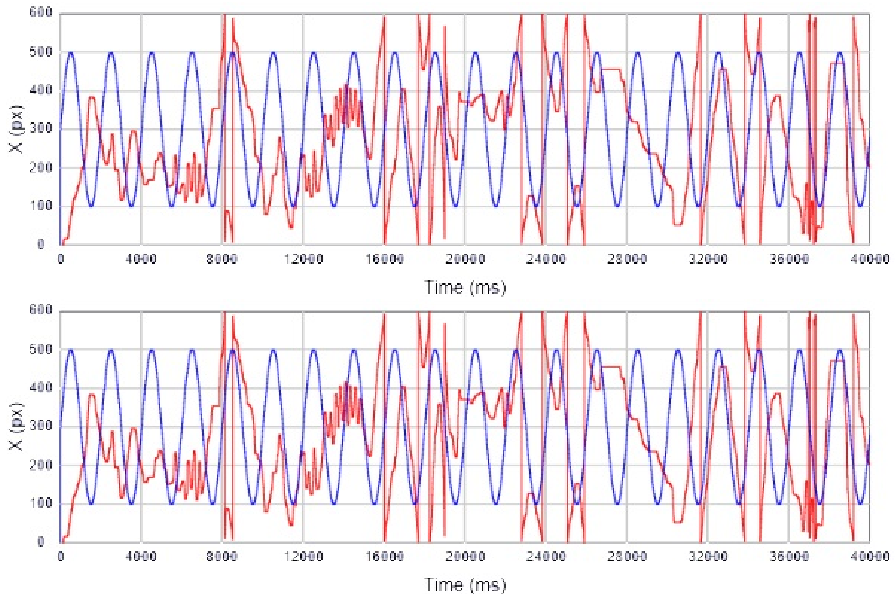

Supplementary Figure 4. Traces of a trial between human vs. 'oscillatory agent' (upper) and (b) human vs. 'shadow agent' (bottom). The red trace corresponds to the human, and the blue ones to the virtual agents.

The 'oscillatory agent' was programmed to deploy a sinusoidal behavior (describing a sinusoidal trajectory of 0.5 Hz and 200 pixels of amplitude), predictable and deterministic. On the other hand, the 'shadow agent' was able to show an irregular pattern because it consisted of the 'shadow image' of the participant (i.e., a bot that generates a movement strictly identical to the participant trajectory but delayed 400 ms. in time and 125 pixels in space).

2 STATISTICAL ANALYSIS

A linear mixed effect model is a multivariate linear regression model which allow us to take into account the fact that we have different players and repeated measures for each player (**Pinheiro and Bates**, 2000).

Model fitting and estimates were obtained with the *nlme library* (**Pinheiro et al.**, 2013) in R (**Team**, 2013). These linear mixed-effects models represent the relationship between the appropriate dependent variable in each of the studies and the type of opponent. Basic assumptions of the models have been checked throughout: as stated before, the potential correlation of repeated measures within players is accounted by means of the mixed-effect models themselves; multicollinearity diagnostics are not necessary as the models are assessed in order to determine how closely these follow the normal distribution and it is concluded that no transformations of the dependent variable are necessary.

Descriptive statistics according to type of opponent and summaries of the main results of the mixed-effect models are given in the table below (Table 1). All models are fitted with an intercept.

Supplementary Table 1. Mean and standard deviation of the performed analyses according to the different type of opponent.

	**Human**	**Oscillatory**	**Shadow**
**Collisions**			
**Number of coll.**	45.79 ± 27.45	37.34 ± 20.68	42.92 ± 37.70
**Colls. 0.25 s**	0.39 ± 0.18	0.15 ± 0.12	0.38 ± 0.19
**Colls. 0.50 s**	0.55 ± 0.16	0.27 ± 0.17	0.71 ± 0.10
**Colls. 1 s**	0.76 ± 0.12	0.63 ± 0.14	0.80 ± 0.11
**Colls. 2 s**	0.90 ± 0.09	0.94 ± 0.06	0.86 ± 0.10
**Fractality**			
β **Interaction**	0.65 ± 0.40	1.51 ± 0.29	0.33 ± 0.47
β **Player**	0.70 ± 0.58	0.49 ± 0.53	0.58 ± 0.49
β **Opponent**	0.79 ± 0.58	2.35 ± 0.22	0.59 ± 0.52
**Multifractality**			
Δh **Interaction**	4.23 ± 1.67	1.31 ± 0.31	3.10 ± 1.77
Δh **Player**	3.40 ± 1.88	2.80 ± 1.26	3.43 ± 2.22
Δh **Opponent**	3.33 ± 1.86	1.50 ± 0.46	3.09 ± 1.60

2.1 COLLISIONS ANALYSES

In a first analysis, the dependent variable is the number of collisions. Thereafter, the dependent variable is the percentage of collisions that are followed by a second collision within a certain period of time. Four time intervals (0.25, 0.5, 1 and 2 seconds) are considered as dependent variables for the corresponding models. Results for the resulting five models can be found below.

The number of collisions (Table 2) gives a poor overall discrimination value for the type of opponent parameter ($p = 0.496$). As well, a detailed view shows poor discrimination between humans and agents.

Supplementary Table 2. Results for the number of collisions.

**Random effects**	**Covariance**					
**Player**	144.752					
**Game**	674.979					
**Fixed effects**	**Estimate**	**Std.Error**	**t-value**	**p-value**	**Lower 95% C.I.**	**Upper 95% C.I.**
**Human (Interc.)**	45.282	5.215	8.683	0.0000	34.926	55.639
**Oscill. agent**	-7.173	6.040	-1.187	0.2381	-19.168	4.823
**Shadow agent**	-2.891	6.533	-0.443	0.6591	-15.865	10.081
**ANOVA**			**F-value**	**p-value**		
**Opponent**			0.70504	0.4967		

A time-scale of 0.25 s (Table 3) gives a good overall discrimination value for the type of opponent parameter ($p < 0.0001$). Nevertheless, a detailed view shows discrimination only on humans versus the oscillatory agent ($p < 0.0001$), but not on humans versus the shadow agent ($p = 0.616$).

A time-scale of 0.5 s (Table 4) gives a good overall discrimination value for the type of opponent parameter ($p < 0.0001$). As well, both cases show a good discrimination between humans and agents.

A time-scale of 1 s. (Table 5) gives a good overall discrimination value for the type of opponent parameter ($p < 0.0001$). Nevertheless, a detailed view shows discrimination only on humans versus the oscillatory agent ($p < 0.0001$), but not on humans versus the shadow agent ($p = 0.246$).

Supplementary Table 3. Results for a time-scale of 0.25 s.

Random effects	Covariance					
**Player**	0.006					
**Game**	0.022					
**Fixed effects**	**Estimate**	**Std.Error**	**t-value**	**p-value**	**Lower 95% C.I.**	**Upper 95% C.I.**
**Human (Interc.)**	0.390	0.033	11.978	0.0000	0.325	0.455
**Oscill. agent**	-0.243	0.035	-6.984	0.0000	-0.313	-0.174
**Shadow agent**	-0.019	0.038	-0.503	0.6159	-0.094	0.056
**ANOVA**			**F-value**	**p-value**		
**Opponent**			26.712	< 0.0001		

Supplementary Table 4. Results for a time-scale of 0.5 s.

Random effects	Covariance					
**Player**	0.005					
**Game**	0.019					
**Fixed effects**	**Estimate**	**Std.Error**	**t-value**	**p-value**	**Lower 95% C.I.**	**Upper 95% C.I.**
**Human (Interc.)**	0.553	0.029	19.247	0.0000	0.496	0.610
**Oscill. agent**	-0.278	0.032	-8.628	0.0000	-0.343	-0.215
**Shadow agent**	0.154	0.035	4.412	0	0.085	0.224
**ANOVA**			**F-value**	**p-value**		
**Opponent**			69.722	< 0.0001		

Supplementary Table 5. Results for a time-scale of 1 s.

Random effects	Covariance					
**Player**	0.003					
**Game**	0.013					
**Fixed effects**	**Estimate**	**Std.Error**	**t-value**	**p-value**	**Lower 95% C.I.**	**Upper 95% C.I.**
**Human (Interc.)**	0.762	0.023	32.509	0.0000	0.715	0.808
**Oscill. agent**	-0.121	0.027	-4.526	0.0000	-0.175	-0.068
**Shadow agent**	0.034	0.029	1.169	0.2455	-0.023	0.091
**ANOVA**			**F-value**	**p-value**		
**Opponent**			14.745	< 0.0001		

A time-scale of 2 s. (Table 6) gives a fair overall discrimination value for the type of opponent parameter ($p = 0.003$). As well, both cases show a fair discrimination between humans and agents ($p = 0.05$).

Supplementary Table 6. Results for a time-scale of 2 s.

Random effects	Covariance					
**Player**	0.001					
**Game**	0.006					
**Fixed effects**	**Estimate**	**Std.Error**	**t-value**	**p-value**	**Lower 95% C.I.**	**Upper 95% C.I.**
**Human (Interc.)**	0.903	0.014	65.160	0.0000	0.875	0.930
**Oscill. agent**	0.037	0.018	1.989	0.0496	0.000	0.073
**Shadow agent**	-0.039	0.020	-1.969	0.0519	-0.078	0.000
**ANOVA**			**F-value**	**p-value**		
**Opponent**			6.136	0.0031		

2.2 FRACTAL AND MULTIFRACTAL ANALYSES

According to the methodology explained in the paper, we consider the β index as the dependent variable for analysing the fractality and the spectrum width Δh as the dependent variable for multifractality. For each case (fractal and multifractal) three data sets are analysed: data of the interaction (relative velocity between the player and her opponent), data of the player (absolute velocity of the player) and data of the opponent (absolute velocity of the opponent). Therefore, a total of six models are conducted.

The interaction dataset (Tables 7-8) gives a good overall discrimination value for the type of opponent parameter ($p < 0.0001$), in both the fractal beta index and of the multifractal spectrum width. As well, both cases show a good discrimination between humans and agents.

Supplementary Table 7. Results for the fractal beta index of the interaction dataset .

**Random effects**	**Covariance**					
**Player**	0.015					
**Game**	0.136					
**Fixed effects**	**Estimate**	**Std.Error**	**t-value**	**p-value**	**Lower 95% C.I.**	**Upper 95% C.I.**
**Human (Interc.)**	0.644	0.065	9.913	0.0000	0.515	0.773
**Oscill. agent**	0.865	0.085	10.130	0.0000	0.696	1.035
**Shadow agent**	-0.298	0.092	-3.229	0.0017	-0.481	-0.114
**ANOVA**			**F-value**	**p-value**		
**Opponent**			78.613	< 0.0001		

Supplementary Table 8. Results for the multifractal spectrum width of the interaction dataset.

**Random effects**	**Covariance**					
**Player**	0.276					
**Game**	1.889					
**Fixed effects**	**Estimate**	**Std.Error**	**t-value**	**p-value**	**Lower 95% C.I.**	**Upper 95% C.I.**
**Human (Interc.)**	4.263	0.237	17.982	0.0000	3.793	4.733
**Oscill. agent**	-2.972	0.311	-9.551	0.0000	-3.590	-2.355
**Shadow agent**	-1.210	0.318	-3.801	0.0002	–1.842	-0.579
**ANOVA**			**F-value**	**p-value**		
**Opponent**			258.350	< 0.0001		

No discrimination potential can be concluded from the lack of statistically significant results from modelling the player dataset (Tables 9-10) for both the fractal beta index ($p = 0.27$) and the multifractal spectrum width ($p = 0.26$). Similarly, no statistically significance differences were found between each of the agents and the reference opponent type category, human.

The opponent dataset (Tables 11-12) gives a good overall discrimination value for the type of opponent parameter ($p < 0.0001$), in both the fractal beta index and of the multifractal spectrum width. Nevertheless, a detailed view shows that agent cases only discriminate humans versus the oscillatory agent ($p < 0.0001$), but not humans versus the shadow agent ($p = 0.085$ and $p = 0.46$).

REFERENCES

Pinheiro, J. and Bates, D. (2000), Mixed-Effects models in S and S-PLUS (New York: Springer.)
Pinheiro, J., Bates, D., and DebRoy, S. (2013), nlme: Linear and nonlinear mixed effects models
Team, R. C. (2013), R: A language and environment for statistical computing

Supplementary Table 9. Results for the fractal beta index of the player dataset.

Random effects	Covariance					
**Player**	0.085					
**Game**	0.221					
**Fixed effects**	**Estimate**	**Std.Error**	**t-value**	**p-value**	**Lower 95% C.I.**	**Upper 95% C.I.**
**Human (Interc.)**	0.680	0.111	6.129	0.0000	0.460	0.901
**Oscill. agent**	-0.177	0.109	-1.611	0.1106	-0.395	0.041
**Shadow agent**	-0.048	0.118	-0.410	0.6831	-0.284	0.188
**ANOVA**			**F-value**	**p-value**		
**Opponent**			1.314	0.2738		

Supplementary Table 10. Results for the multifractal spectrum width of the player dataset.

Random effects	Covariance					
**Player**	0.254					
**Game**	3.125					
**Fixed effects**	**Estimate**	**Std.Error**	**t-value**	**p-value**	**Lower 95% C.I.**	**Upper 95% C.I.**
**Human (Interc.)**	3.390	0.275	12.311	0.0000	2.844	3.936
**Oscill. agent**	-0.590	0.396	-1.485	0.1405	-1.379	0.198
**Shadow agent**	0.072	0.408	0.178	0.8594	-0.736	0.881
**ANOVA**			**F-value**	**p-value**		
**Opponent**			1.362	0.2608		

Supplementary Table 11. Results for the fractal beta index of the opponent dataset..

Random effects	Covariance					
**Player**	0.003					
**Game**	0.231					
**Fixed effects**	**Estimate**	**Std.Error**	**t-value**	**p-value**	**Lower 95% C.I.**	**Upper 95% C.I.**
**Human (Interc.)**	0.791	0.070	11.223	0.0000	0.651	0.932
**Oscill. agent**	1.563	0.110	14.270	0.0000	1.345	1.781
**Shadow agent**	-0.207	0.119	-1.741	0.0850	-0.442	0.029
**ANOVA**			**F-value**	**p-value**		
**Opponent**			129.608	< 0.0001		

Supplementary Table 12. Results for the multifractal spectrum width of the opponent dataset.

Random effects	Covariance					
**Player**	0.045					
**Game**	2.336					
**Fixed effects**	**Estimate**	**Std.Error**	**t-value**	**p-value**	**Lower 95% C.I.**	**Upper 95% C.I.**
**Human (Interc.)**	3.336	0.210	15.866	0.0000	2.920	3.753
**Oscill. agent**	-1.830	0.339	-5.398	0.0000	-2.503	-1.158
**Shadow agent**	-0.259	0.349	-0.741	0.4601	-0.952	0.434
**ANOVA**			**F-value**	**p-value**		
**Opponent**			15.2556	< 0.0001		
